# Levo-tetrahydropalmatine attenuates mouse blood-brain barrier injury induced by focal cerebral ischemia and reperfusion: Involvement of Src kinase

**DOI:** 10.1038/srep11155

**Published:** 2015-06-10

**Authors:** Xiao-Wei Mao, Chun-Shui Pan, Ping Huang, Yu-Ying Liu, Chuan-She Wang, Li Yan, Bai-He Hu, Xin Chang, Ke He, Huan-Na Mu, Quan Li, Kai Sun, Jing-Yu Fan, Jing-Yan Han

**Affiliations:** 1 Department of Integration of Chinese and Western Medicine, School of Basic Medical Sciences, Peking University, Beijing, China; 2 Key Laboratory of Microcirculation, State Administration of Traditional Chinese Medicine of China, Beijing, China; 3 Tasly Microcirculation Research Center, Peking University Health Science Center, Beijing, China; 4 Key Laboratory of Stasis and Phlegm, State Administration of Traditional Chinese Medicine of China, Beijing, China

## Abstract

The restoration of blood flow following thrombolytic therapy causes ischemia and reperfusion (I/R) injury leading to blood-brain barrier (BBB) disruption and subsequent brain edema in patients of ischemic stroke. Levo-tetrahydropalmatine (l-THP) occurs in Corydalis genus and some other plants. However, whether l-THP exerts protective role on BBB disrpution following cerebral I/R remains unclear. Male C57BL/6N mice (23 to 28 g) were subjected to 90 min middle cerebral artery occlusion, followed by reperfusion for 24 h. l-THP (10, 20, 40 mg/kg) was administrated by gavage 60 min before ischemia. We found I/R evoked Evans blue extravasation, albumin leakage, brain water content increase, cerebral blood flow decrease, cerebral infarction and neurological deficits, all of which were attenuated by l-THP treatment. Meanwhile, l-THP inhibited tight junction (TJ) proteins down-expression, Src kinase phosphorylation, matrix metalloproteinases-2/9 (MMP-2/9) and caveolin-1 activation. In addition, surface plasmon resonance revealed binding of l-THP to Src kinase with high affinity. Then we found Src kinase inhibitor PP2 could attenuate Evans blue dye extravasation and inhibit the caveolin-1, MMP-9 activation, occludin down-expression after I/R, respectively. In conclusion, l-THP attenuated BBB injury and brain edema, which were correlated with inhibiting the Src kinase phosphorylation.

Ischemic stroke is a condition in clinic that causes severe long-term disability or death. Thrombolytic therapy after stroke may result in ischemia and reperfusion (I/R) injury manifesting blood-brain barrier (BBB) disruption leading to brain edema and hemorrhagic transformation, which is one of the life-threatening complications after acute ischemic stroke. However, so far no effective strategy is available for I/R-induced BBB disruption^1^,^2^.

The BBB is composed of microvascular endothelial cells, basement membrane (BM), astrocyte endfeet, and pericytes. The endothelial cells are the first-line of the defense between the blood and the brain^3^. The permeability of the endothelial barrier is regulated by two different routes, one is transcellular pathway which is via caveolae-mediated vesicular transport, the other is paracellular pathway controlled by interendothelial junctions. As the principal marker of caveolae, caveolin-1 is required for the caveolae-mediated transcytosis of albumin across endothelial cells^4^,^5^. Tight junction (TJ) between adjacent endothelial cells plays critical roles in the BBB disruption during ischemic stroke. The TJ proteins claudin-5, occludin, and zonula occludens-1 (ZO-1) have been reported to participate in the ischemic BBB injury^6^,^7^,^8^. Activation of myosin light chain kinase (MLCK) by phosphorylated Src kinase has been shown to increase actin-myosin interaction and subsequent TJ proteins ZO-1 and occludin redistribution^6^,^9^,^10^,^11^. Additionally, inhibition of the Src kinase phosphorylation is known to attenuate caveolae-mediated transcytosis of albumin^12^.

As an inherent part of BBB, BM is composed of extracellular matrix (ECM) molecules such as type IV collagen, laminins, fibronectin, heparan sulfates, and proteoglycans^13^. Matrix metalloproteinases (MMPs), particularly MMP-2 and MMP-9, play a critical role in BBB injury in stroke by degrading the ECM components of the BM^14^,^15^. Meanwhile, upregulated expression of MMP-2/9 has been shown to mediate the degradation of several TJ proteins including occludin, claudin-5, and ZO-1^16^,^17^.

Levo-tetrahydropalmatine (l-THP) occurs in Corydalis genus, which is included in Cerebralcare Granule, a newly developed compound Chinese medicine for treatment of migraine and vertigo. Previous studies have shown that l-THP could attenuate cerebral infraction and inhibit the neuron apoptosis by modulating the expression of heat shock protein 70 (HSP70), bcl-2 and bax after acute global cerebral I/R^18^,^19^,^20^. However, the effect of l-THP on BBB injury after focal cerebral I/R has not been explored. The study was designed to investigate the role of l-THP on BBB injury after I/R, with particularly addressing the involvement of Src kinase.

## Results

### l-THP attenuates CBF in the ischemic core, reduces infarction size and improves neurological deficits

**Figure 1:**
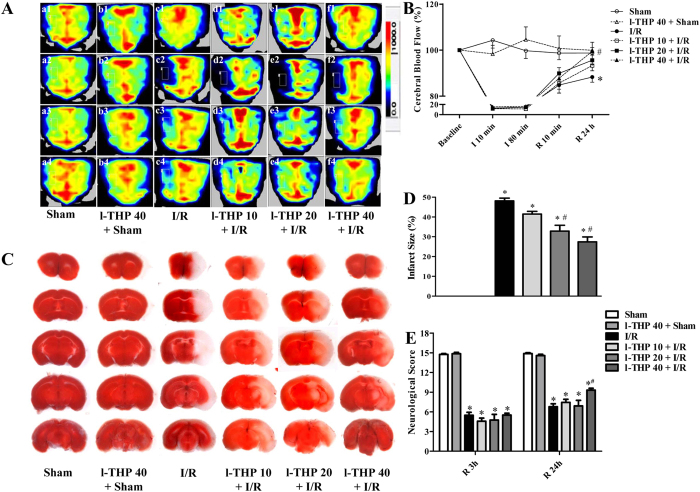
l-THP attenuates infract size, neurological deficits and CBF. (**A**) The representative images of cerebral blood flow of ipsilateral cortex in different groups at different time points. The magnitude of CBF is represented by different colors, with blue to red denoting low to high. 1, 2, 3, and 4 represents baseline, ischemia 10 min, reperfusion 10 min, and reperfusion 24 h, respectively. (**B**) Quantitative analysis of CBF in different groups at different time points. (**C**) Representative images of brain coronal slices stained by TTC in different groups, wherein normal brain tissue is stained red, while the infarct lesion remains unstained. (**D**) Quantitative analysis of infarct size in different groups. (**E**) Neurological scores of animals in different groups evaluated at reperfusion 3 h (R 3 h) and reperfusion 24 h (R 24 h). Values are the mean ± SEM. *P < 0.05 vs. Sham group, #P < 0.05 vs. I/R group.

We assessed the CBF in ischemic core of mouse brain in different groups using a laser Doppler perfusion image system, and the representative images of different groups at different time points are presented in Fig. 1A, while the quantification of the results depicted in Fig. 1B. The result showed that no significant difference in CBF was observed in Sham and l-THP 40 + Sham groups over the entire experiment. In contrast, ischemia for 10 min reduced CBF to a level lower than 20% of baseline (Fig. 1A, c2-f2, Fig. 1B), which returned to a lever higher than 70% of baseline after 10 min reperfusion (Fig. 1A, c3-f3, Fig. 1B) in all I/R groups without significant difference detected among groups. Of notice, at 24 h after reperfusion, CBF was significantly higher in l-THP 40 + I/R group than that in I/R group. l-THP at 10 mg/kg and 20 mg/kg improved CBF as well at 24 h after reperfusion but without statistic significance compared with I/R only group.

TTC staining was used to assess cerebral infarct and the result is shown in Fig. 1C,D. As expected, brain infarct size significantly increased in I/R group when compared with Sham group. Noticeably, the I/R-elicited cerebral infarct was reduced by l-THP 20 and l-THP 40 treatment significantly, but not by l-THP 10 treatment.

Neurological function was scored at 3 h and 24 h after reperfusion. As shown in Fig. 1E, l-THP at 40 mg/kg significantly improved neurological deficit scores compared with the I/R group at 24 h after reperfusion, but not at 3 h after reperfusion or lower doses.

Taken together, these findings demonstrated that l-THP attenuated the I/R-elicited CBF decrease, brain infarction and neurological deficits, with the higher dose of l-THP (40 mg/kg) being more efficient. The dose of 40 mg/kg was thus selected as an optimal dose of l-THP and applied in all subsequent experiments.

### l-THP reduces albumin leakage, Evans blue extravasation and brain water content in mice

**Figure 2:**
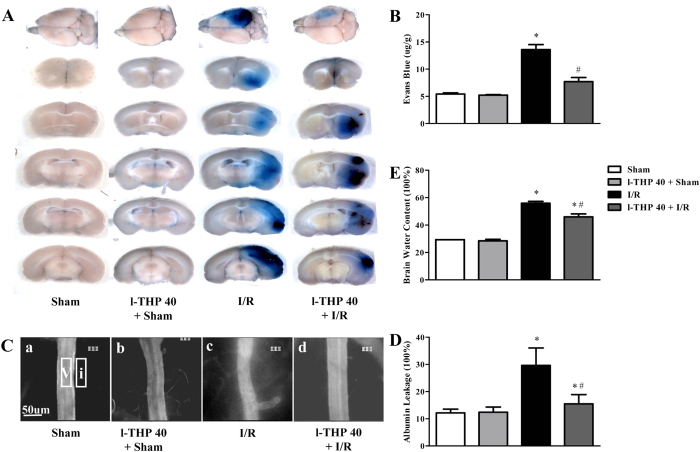
l-THP reduces Evans blue extravasation, albumin leakage and brain water content in mice. (**A**) The representative pictures of Evans blue extravasation in right brains of various groups. (**B**) The quantitative analysis of Evans blue leakage. (**C**) Representative images of albumin leakage from venules in all groups. Rectangles represent the areas for determination of fluorescence. V: cerebral venule. I: Interstitial tissue. (**D**) Statistic analysis of albumin leakage. (**E**) Brain water content. Values are the mean ± SEM. ^*^P < 0.05 vs. Sham group, # P < 0.05 vs. I/R group.

BBB permeability was tested by Evans blue dye extravasation and albumin leakage at the right cerebral hemisphere of mice in various groups. As shown in Fig. 2A,B, Evans blue dye extravasation in I/R group was markedly increased at 24 h after reperfusion compared with the Sham group, which was attenuated significantly by l-THP treatment. Likewise, the albumin leakage in I/R group increased significantly at 24 h after reperfusion compared with the Sham group, which was blunted apparently by l-THP treatment (Fig. 2C, D). Increased BBB permeability may lead to brain edema, thus we further assessed brain water content in different groups. As expected, I/R increased brain water content significantly, while treatment with l-THP prevented this increase significantly (Fig. 2E).

### l-THP attenuates the alteration in ultrastructure of microvessels

**Figure 3:**
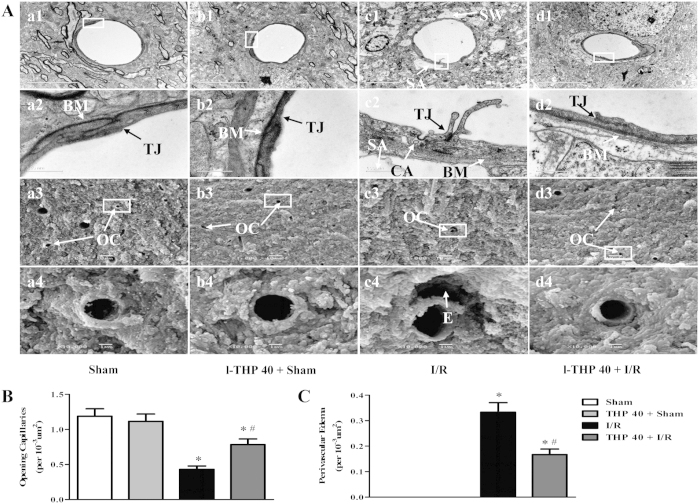
l-THP improves ultrastructure of microvessels in the ipsilateral cerebral cortex of mice after I/R. (**A**) The upper two panels are the representative transmission electron micrographs of capillaries in the cerebral cortex in different groups, while lower two panels show the scanning electron micrographs of the cerebral cortex fractured face. The micrographs in 2 and 4 are the high magnification of the area inside the boxes in 1 and 3, respectively. SA: swelling astroglial process; OC: opening capillaries; E: perivascular edema; TJ: tight junction; BM: basement membrane; CA: caveolae. (**B**) Quantitative analysis of the number of opening capillaries, evaluated in 6 randomly selected scanning electron micrographs. (**C**) Quantitative analysis of the number of perivascular edema, statistics method is same as (**B**). ^*^P < 0.05 vs. Sham group, #P < 0.05 vs. I/R group.

Transmission electron microscopy clearly identified the structures of cerebral microvasculature in the cortex, as shown in Fig. 3Aa1-d1 and a2-d2. The cerebral microvasculature in Sham group displayed a normal structure with smooth inner surface and closely connected endothelial cells via tight junction. No swelling structure was observed surrounding the microvessels. In contrast, the I/R group showed a remarkable alteration in the microvessels at 24 h after reperfusion, such as ruptured and discontinuous BM (Fig. 3Ac2), opened TJ (Fig. 3Ac2), more caveolae in the cytoplasm of capillary endothelial cells (Fig. 3Ac2), as well as evident swelling perivascular astrocyte end feet (Fig. 3Ac1 and c2), a morphological manifestation consistent with brain edema. A further examination using scanning electron microscopy revealed an obvious reduction in the number of opening microvessels and perivascular edema (Fig. 3Ac3 and d3) in I/R group compared with Sham group. However, l-THP treatment apparently attenuated all the alterations in microvessels induced by I/R. The quantification of the number of opening capillaries and perivascular edema depicted in Fig. 3B,C.

### l-THP protects against the decrease in TJ proteins expression and caveolin-1 activation

**Figure 4:**
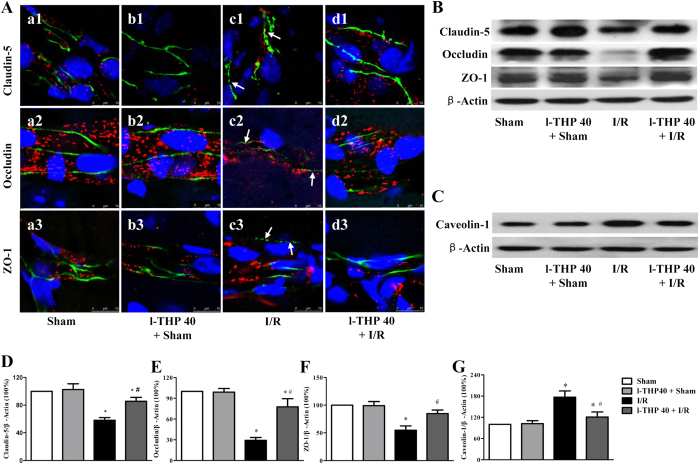
l-THP protects the decrease in TJ proteins expression and the activation of caveolin-1. (**A**) Representative immunofluorescence confocal images in different groups of claudin-5 (a1-d1, green), occludin (a2-d2, green) and ZO-1 (a3-d3, green) localized at the periphery of endothelial cells with marker vWF (red). (**B**) Representative western blots of claudin-5, occludin and ZO-1. (**C**) Representative western blots of caveolin-1 protein. (**D**–**G**) Quantitative analysis of claudin-5, occludin, ZO-1 and caveolin-1. Values are the mean ± SEM. ^*^P < 0.05 vs. Sham group, # P < 0.05 vs. I/R group.

To gain insight into the rationale behind the role of l-THP in maintaining BBB integrity, vascular endothelial TJ proteins were examined for various groups by confocal microscopy and western blot (Fig. 4A,B). Confocal microscopy revealed that claudin-5 localized between endothelial cells as continuous lines in Sham and l-THP 40 + Sham groups (Fig. 4Aa1 and 4Ab1). In I/R group, these continuous distributions were disrupted apparently after 24 h reperfusion, becoming dotted lines, concomitant with reduction in immune staining, indicating an decrease in the tight junction proteins claudin-5 expression (Fig. 4A c1). Interestingly, this decrease was restored evidently by l-THP treatment (Fig. 4A d1). These results were confirmed by western blot (Fig. 4B,D). Similar results were obtained for occludin and ZO-1 (Fig. 4A,B,E,F).

Western blot was used to analyze the caveolin-1 protein expression (Fig. 4C,G), showing a marked increase in caveolin-1 protein level in the ischemic hemisphere of MCAO mice at 24 h after reperfusion compared with Sham group. Impressively, l-THP treatment significantly attenuated the caveolin-1 protein expression increase induced by I/R.

### l-THP inhibits the expression of MMP-2/9 and Src/MLCK/MLC signaling pathway to attenuated BBB injury

**Figure 5:**
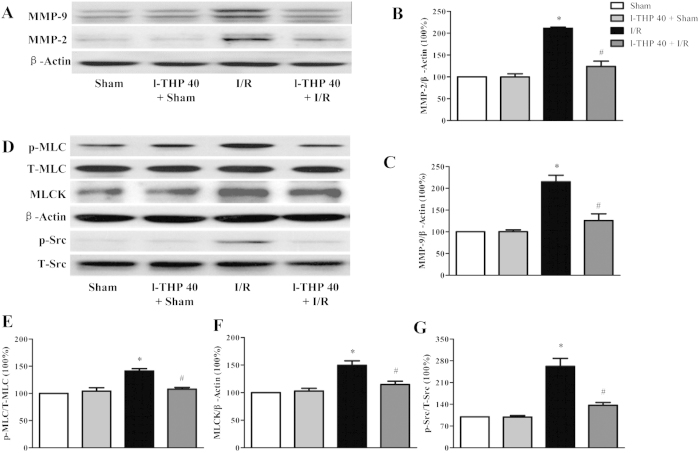
l-THP inhibits the expression of MMP-2/9 and Src/MLCK/MLC signaling pathway after I/R. (**A**) Representative western blots of MMP-2/9 in different groups. (**B**) and (**C**) Quantitative analysis of MMP-2 and MMP-9, respectively. (**D**) Representative western blots of p-Src, MLCK and p-MLC. (**E**–**G**) Quantitative analysis of p-MLC, MLCK and p-Src, respectively. Values are the mean ± SEM. ^*^P < 0.05 vs. Sham group, # P < 0.05 vs. I/R group.

Upregulated expressions of MMP-2/9 have been shown to mediate the degradation of BM and several TJ proteins. Firstly, we using western blot determined whether l-THP is able to inhibit the expression of MMP-2/9 after I/R. As shown in Fig. 5A–C, there was a marked increase in MMP-2 and MMP-9 protein levels in the ischemic hemisphere of MCAO mice at 24 after reperfusion compared with sham group. Impressively, l-THP treatment significantly attenuated the increase in the expression of MMP-2 and MMP-9 induced by focal I/R.

Activation of MLCK by phosphorylated Src kinase has been shown to lead to increased actin-myosin interaction and subsequently increased paracellular permeability. In the present study, a remarkable increase in the phosphorylation of Src kinase and MLC proteins and the expression of MLCK was observed in I/R group at 24 h after reperfusion compared with Sham group, which were significantly attenuated by l-THP treatment (Fig. 5D through 5G).

Taken together, these results suggested that l-THP attenuated the BBB injury after I/R at least partly via inhibiting the expression of MMP-2/9 and the activation of Src/MLCK/MLC-signaling pathway.

### l-THP binds to Src in a dose-dependent manner

**Figure 6:**
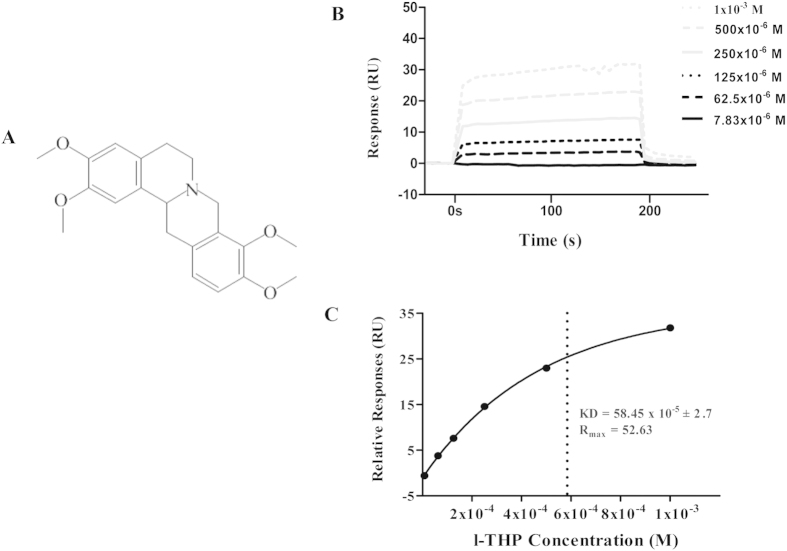
The chemical structure of l-THP and the binding kinetic of l-THP to Src. (**A**) The chemical structure of l-THP. (**B**) The binding kinetic of l-THP to Src at different concentrations assessed by SPR. (**C**) Affinity derived by fitting the kinetic data to a 1:1 Langmuir binding model utilizing global fitting algorithms for determination of the dissociation rate (KD) of biomolecule interactions.

In view of the critical role of Src in l-THP action, we determined the interaction between Src and l-THP by using SPR. As shown in Fig. 6B,C, l-THP bound to Src in a dose-dependent manner. The KD is 58.45 × 10^−5^ ± 2.7 as obtained by SPR, indicating the likelihood of directly binding of l-THP to Src kinase.

### PP2 inhibits the Src kinase phosphorylation after I/R to attenuate BBB injury

**Figure 7:**
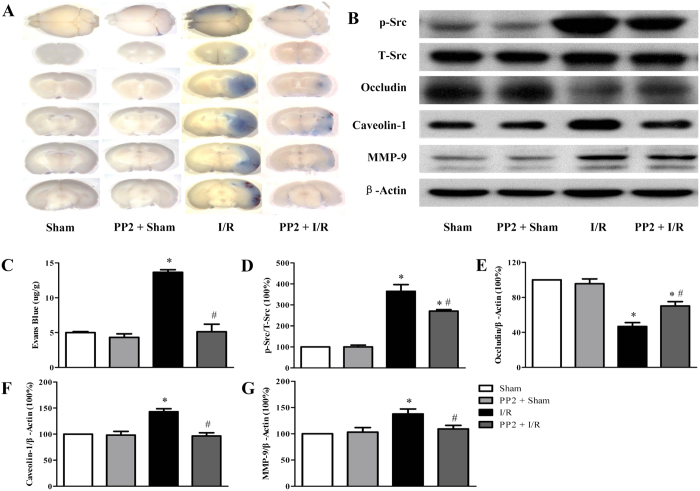
PP2 inhibits the Src kinase phosphorylation after I/R to attenuate BBB injury. (**A**) The representative pictures of Evans blue extravasation in right brains of various groups. (**B**) Representative western blots of p-Src, occluding, caveolin-1 and MMP-9, respectively. (**C**) The quantitative analysis of Evans blue leakage. (**D**–**G**) Quantitative analysis of p-Src, occludin, caveolin-1 and MMP-9. Values are the mean ± SEM. P < 0.05 vs. Sham group, # P < 0.05 vs. I/R group.

In order to prove Src kinase phosphorylation as the up-stream of caveolin-1, TJ proteins and MMP-2/9, we tested the effect of Src kinase inhibitor PP2 *in vivo*. As shown in Fig. 7A,C, PP2 attenuated Evans blue dye extravasation in PP2 + I/R group compared with I/R group. Then we used western blot to detect whether PP2 could inhibit the caveolin-1, MMP-9 activation and occludin down-expression. We found that PP2 inhibited the Src kinase phosphorylation after I/R (Fig. 7B,D), significantly. Meanwhile, PP2 inhibited the caveolin-1, MMP-9 activation and occludin down-expression (Fig. 7B,E–G), respectively.

## Discussion

The present study demonstrated that l-THP attenuated BBB injury and brain edema induced by focal cerebral I/R, and meanwhile, reduced brain infarct size and neurological deficits. Furthermore, l-THP displayed potential to inhibit the decrease in TJ proteins expression and caveolin-1 activation after cerebral I/R, and blunt the expression of MMP-2/9 and Src/MLCK/MLC-signaling pathway.

l-THP is an alkaloid (Fig. 6A) found in the Corydalis genus (Yan Hu Suo) and some other plants such as Stephania rotunda. l-THP is a blocker of voltage-activated L-type calcium channel activated potassium channels, and used in clinic mainly as anxiolytic and sedative drugs^21^,^22^. l-THP is included in Cerebralcare Granule^@^ as an active ingredient, potentially contributable to its effect on migraine and vertigo. Studies in animals have shown the beneficial role of l-THP in heart disease and liver injury^21^,^23^. Most of the experimental studies so far have focused on the mechanisms behind its anxiolytic and sedative effect, while that regarding the role of l-THP in cerebral I/R injury is limited. Few available study showed that l-THP could attenuate cerebral infraction and inhibit the neuron apoptosis by modulating the expression of HSP70, bcl-2 and bax induced by acute global cerebral I/R^18^,^19^,^20^. In line with these results, the present study using MCAO as a model revealed that l-THP is able to protect against cerebral I/R-induced infarct and neurological deficits, providing further evidence supporting l-THP as a potential strategy to cope with cerebral I/R injury in clinic. Nevertheless, clinic translation of l-THP for management of cerebral I/R injury needs more studies, particularly in the dosage and timing of administration.

Arterial recanalization, for example by thrombolytic therapy, and subsequent reperfusion have demonstrated as an effective measure to restore the brain function if performed shortly after acute ischemic stroke^1^,^2^. However, arterial recanalization does not always benefit stoke patients. In some cases, sudden tissue reperfusion may be deleterious, leading to BBB disruption and hemorrhagic transformation or massive brain edema due to the so-called ‘reperfusion injury’^24^. Remedy targeting toward BBB disruption after cerebral I/R is thus expected to be a strategy to extent the efficiency of recanalization treatment of ischemic stroke. The present study showed the potential of l-THP to attenuate BBB disruption caused by I/R, as evidenced by assessment of albumin leakage, Evens blue extravasation and brain water content. This result highlights the preservation of BBB as the mechanism underlying the beneficial role of l-THP in cerebral I/R injury. Furthermore, l-THP blunted all the alterations in caveolin-1 activation, TJ proteins down-expression, MMP-2/9 and Src kinase activation, the major contributors to BBB, after cerebral I/R, highly suggesting that l-THP exerts its effect by acting at some target upstream these determinants. We postulate that Src may serve as such a target.

Src-family kinases are signaling proteins involved in a diverse array of cellular processes, including BBB regulation. Src kinase has been shown to modulate MMPs thus participate in BBB regulation^25^. Study revealed that Src is involved in tyrosine phosphorylation of occludin attenuating its interaction with ZO-1, ZO-2, and ZO-3^26^. Moreover, increasing evidence demonstrates the role of Src in caveolin-1 phosphorylation and activation^27^. In view of the pivotal role of Src kinase in regulating BBB, development of medicine targeting Src kinase to attenuate I/R-induced BBB disruption has received attention. In the present study, we found that THP attenuated the phosphorylation of Src kinase. Moreover, assessment by SPR demonstrated that l-THP bound to Src kinase in a dose-dependent manner with the KD 58.45 × 10^−5^ ± 2.7. These data highly support the speculation that l-THP binds Src kinase as its target initiating its protective effect on BBB disruption and cerebral I/R injury. Then we found that Src inhibitor PP2 inhibited the caveolin-1, MMP-9 activation and occludin down-expression. Nevertheless, the detail of the mechanism for l-THP interaction with Src requires further study.

In conclusion, the present study demonstrated the potential of l-THP to protect against cerebral I/R-induced BBB injury and attenuate brain edema and infarct. It is highly likely that this protective effect of l-THP initiates with its binding to Src kinase thus inhibiting the phosphorylation of Src kinase. These results provide evidence supporting l-THP as novel prophylaxis for cerebral I/R injury in stroke.

## Materials and methods

### Animals

Male C57BL/6N mice (23 to 28 g) were purchased from the Animal Center of Peking University Health Science Center (Beijing, certificate no. SCXK 2006-0008). The animals were housed in cages at 22 °C ± 2 °C and humidity of 40% ± 5% under a 12-hour light/dark cycle. The experimental procedures were carried out in accordance with the European commission guidelines (2010/63/EU). Animal handling was approved by the Committee on the Ethics of Animal Experiments of the Health Science Center of Peking University (LA2011–38).

### Focal ischemia-reperfusion model and experimental groups

Focal ischemia was induced using middle cerebral artery occlusion (MCAO) method which has been described previously^28^. Briefly, mice were anesthetized with isoflurane in 70%N_2_O/30%O_2_, the left common and external carotid arteries were isolated and ligated. A microvascular clip was placed on the internal carotid artery. A 6-0 nylon monofilament coated with silicon resin was introduced through a small incision in the external carotid artery and advanced to a position 9 mm distal from the carotid bifurcation for occlusion the origin of middle cerebral artery. Reperfusion was initiated by withdrawal of the monofilament after 90 min occlusion. Anesthesia was discontinued and animals were placed back into their cages. Rectal temperature was maintained at 37 ± 0.5 °C through a thermostat-controlled heating pad. 24 h later, mice were deeply re-anesthetized and decapitated. Brains were removed for experiment.

A total of 188 mice were included and randomly divided into Sham group (Sham, n = 39), l-THP 40 + Sham group (l-THP 40 + Sham, n = 39), PP2 + Sham group (PP2 + Sham, n = 8), I/R group (I/R, n = 39), l-THP 10 + I/R group (l-THP 10 + I/R, n = 8), l-THP 20 + I/R group (l-THP 20 + I/R , n = 8), l-THP 40 + I/R group (l-THP 40 + I/R, n = 39), PP2 + I/R group (PP2 + I/R, n = 8) (see Table 1 for further details). In the Sham, PP2 + Sham and l-THP 40 + Sham, mice were operated in the same way as that in I/R, but without occlusion of middle cerebral artery. In the l-THP 40 + Sham, l-THP 10 + I/R, l-THP 20 + I/R and l-THP 40 + I/R, l-THP (10, 20, 40 mg/kg) were administrated by gavage 60 min before ischemia, while 10 mg/kg was sedative dose used in clinical. In the PP2 + Sham and PP2 + I/R group, PP2 (60 ug/kg) were administrated by gavage 60 min before ischemia. The mice in Sham and I/R were given equivalent volume of saline in the same manner.

### Cerebral blood flow measurement

Cerebral blood flow (CBF) was measured using Laser Doppler perfusion image system (PeriScan PIM3 System; PERIMED, Stockholm, Sweden) as previously described^29^ with some modifications. Briefly, after retracing the scalp, a low-powered He-Ne laser beam was directed by computer-controlled optical scanner over the exposed cortex with the scanner head positioned in parallel to the cerebral cortex at a distance of 18.5 cm. The CBF was measured before the cerebral ischemia (baseline), 10 min and 80 min after ischemia, 10 min and 24 h after reperfusion, respectively. A color-coded image denoting specific relative perfusion levels was displayed on a video monitor and the ischemia core area in all images was evaluated with the software LDPIwin 3.1 (PeriScan PIM3 System; PERIMED, Stockholm, Sweden). Only those mice that had a CBF lower than 20% of baseline at 10 min and 80 min after ischemia and higer than 70% of baseline at 10 min after reperfusion were considered as successful induction of focal cerebral I/R.

### Infarct size and neurological deficits

Infarct size was measured using 2% 2,3,5-triphenyltetrazolium chloride (TTC) staining as described previously^29^. Briefly, mouse brains were removed at 24 h after MCAO and sliced into 5 coronal sections (2 mm thick). Then the slices were stained with 2% TTC for 15 minutes at 37 °C and scanned, the infarct area was estimated by Image J (Bethesda, MD, USA) software. The infarct volume was calculated using a formula^30^ as follows: 100 × (contralateral hemisphere volume—non-infarct ipsilateral hemisphere volume)/contralateral hemisphere volume.

Animals were evaluated for neurological deficits at 3 h and 24 h after MCAO as previously described^31^. The scoring system included five indexes: spontaneous activity over a 3 min period (0–3), symmetry of movement (0–3), open-field path linearity (0–3), beam walking on a 3 × 1 cm beam (0–3), and response to vibrissae touch (1–3), with score 15 being normal while 0 being death.

### Albumin leakage, Evans blue extravasation and brain water content

BBB injury after I/R was quantitatively evaluated by albumin leakage^32^ and Evans blue dye^33^.

For observation of albumin leakage, a noninvasive method was applied as previously described^34^. Briefly, after anesthetized, the mouse skull was ground down with a cranial drill over the right parietooccipital cortex, a location corresponding to the margin of the MCA territory. The cerebral venules ranging from 35 to 45 μm in diameter and 200 μm in length were selected under a fluorescence microscope (BX51WI, Olympus, Tokyo, Japan). Ten minutes before observation, the mouse was intravenously injected with 50 mg/kg fluorescein isothiocyanate (FITC)-albumin (Sigma-Aldrich, St Louis, MO, USA) through femoral vein. Fluorescence signal (excitation wave length at 420 to 490 nm, emission wave length at 520 nm) was acquired using a super-sensitive CCD camera (USS-301, UNIQ Vision Inc, Santa Clara, CA, USA). The fluorescence intensities of FITC-albumin in the venules (Iv) and the perivenular interstitial area (Ii) were assessed with image J (Bethesda, MD, USA) software. Albumin leakage was presented as Ii/Iv^32^.

Four percent of Evans blue dye (Sigma-Aldrich, StLouis, MO, USA) in 0.9% saline (2 mL/kg) was injected into the right femoral vein. 3 hrs later, mice were transcardially perfused, and ischemic hemispheres were removed, homogenized in 1 mL of 50% trichloroacetic acid and centrifuged. The supernatant was diluted four-fold with ethanol. A fluorescent plate reader (620 nm excitation and 680 nm emission) was used to determine dye concentrations. The amount of extravasated Evans blue was expressed as nanograms per ischemic hemisphere.

Brains water content was measured as described^32^. Briefly, brains were quickly separated into the left and right cerebral hemispheres and weighed (wet weight). Brain specimens were then dried in an oven at 120 °C for 48 h and weighed again (dry weight). The percentage of water content was calculated as (wet weight−dry weight)/wet weight × 100.

### Ultrastructure of microvessls

The mouse brain was perfused for 40 min with a fixative consisted of 4% formaldehyde and 2% glutaraldehyde in 0.1 mol/L phosphate buffer at a speed of 3 mL/min. For transmission electron microscopy, a coronal slice of approximately 1 mm thick through the cortex penumbra area was taken. The slice was placed in fresh prepared 3% glutaraldehyde overnight at 4 °C. After rinsing with 0.1 mol/L phosphate buffer for 3 times, the tissue block was post-fixed in 1% osmium tetroxide and processed for ultrathin section and examined in a transmission electron microscope (JEM 1230, JEOL, Tokyo, Japan). For scanning electron microscopy, the samples were cut into blocks and placed in the freshly prepared glutaraldehyde for 2 h, rinsed with 0.1 mol/L phosphate buffer, and then post-fixed in 1% osmium tetroxide in 0.1 mol/L phosphate buffer for 2 h. The specimens were processed as routing and examined under a scanning electron microscope (JSM-5600LV, JEOL, Tokyo, Japan). The number of opening capillaries and perivascular edema was estimated by Image J (Bethesda, MD, USA) software.

### Immunofluorescence staining

Coronal fresh frozen sections were sliced in 10 μm thick using a cryostat (CM1800, Leica, Bensheim, Germany). After washed with PBS for three times and blocking with 3% normal goat serum at room temperature for 0.5 h, the slices were incubated with primary antibodies diluted in PBS overnight at 4 °C. The primary antibodies were applied as follows: mouse anti-claudin-5 (1:100, Invitrogen, Camarillo, CA, USA), mouse anti-occludin (1:50, Invitrogen, Camarillo, CA, USA), mouse anti-ZO-1 (1:50, Invitrogen, Camarillo, CA, USA), and rabbit anti-vWF (1:100, Millipore, Temecula, CA, USA). Then the brain sections were incubated with Dylight 488-labeled goat anti-rabbit IgG (KPL, Gaithersburg, MD, USA) and Dylight 549-labeled goat anti-mouse IgG (KPL, Gaithersburg, MD, USA), for 2 h at room temperature. Hoechst 33342 (Molecular Probes) was applied to stain the nuclei. The brain sections were mounted and cover slipped, and photographed under a laser scanning confocal microscope (TCS SP5, Leica, Mannheim, Germany).

### Western blotting

Western blot analysis was performed as described previously^29^. Briefly, protein concentrations were estimated by the Bradford method, equal amount of protein (100 μg/lane) was diluted in 10 × sample buffer, boiled, and loaded onto 12% SDS-PAGE gels and transferred to a nitrocellulose membrane (Hybond-C, Amersham Biosciences, USA). The membranes were incubated overnight at 4 °C with the primary antibodies against β-actin, occludin, ZO-1, MLC, MLCK, MLC (S20) (Abcam, Cambridge, UK), claudin-5, MMP-2, MMP-9 (Santa Cruz Biotechnology, Santa Cruz, USA), caveolin-1, Src, caveolin-1 and Src (Tyr416) (Cell Signaling, Beverly, Massachusetts, USA). Then the membranes were washed with TBST and incubated with the respective horseradish peroxidase-conjugated secondary antibodies at a 1:5000 dilution for 60 min at room temperature. The protein bands were detected by enhanced chemiluminescence, and the band intensities were quantified by densitometry and expressed as mean area density using the image J (Bethesda, MD, USA) software.

### Surface plasmon resonance

Carboxymethylated 5 sensor chip was docked into the BIAcore 3000 (GE Healthcare, Buckinghamshire, UK). Human recombinant Src (Abcam, Cambridge, UK) was immobilized on CM5 sensor chip by injecting 40 μl of Src (40 μg/ml in 10 mM sodium acetate, pH 4.5) at the rate of 5 μl/min. l-THP was prepared as a 20 mM solution in running buffer before the experiment, and two fold diluted by running buffer into 1000 uM, 500 uM, 250 uM, 125 uM, 62.5 μM, 31.3 μM, 15.65 uM, 7.83 uM before injection. Analytes were injected at 30 μl/min over Src and control sensor chip. Equilibrium dissociation (KD) was calculated by fitting a 1:1 Langmuir model using the BIA evaluation 4.1 software (GE Healthcare, Buckinghamshire, UK).

### Statistical Analysis

All data were expressed as mean ± SEM. Statistics were performed with analysis of variance (ANOVA) with Bonferroni post hoc correction when needed for multiple comparisons. A value of P < 0.05 was considered statistically significant.

## Additional Information

**How to cite this article**: Mao, X.-W. *et al.* Levo-tetrahydropalmatine attenuates mouse blood-brain barrier injury induced by focal cerebral ischemia and reperfusion: Involvement of Src kinase. *Sci. Rep.* **5**, 11155; doi: 10.1038/srep11155 (2015).

## Figures and Tables

**Table 1:** The number of animals for different experimental groups and various parameters.

	**CBF & TTC & NSS**	**EB**	**Micro & BWC**	**EM**	**IHC**	**WB**	**Total**
**Sham**	8	8	8	3	8	4	39
**l-THP40 + Sham**	8	8	8	3	8	4	39
**PP2 + Sham**		4				4	8
**I/R**	8	8	8	3	8	4	39
**l-THP10 + I/R**	8						8
**l-THP20 + I/R**	8						8
**l-THP40 + I/R**	8	8	8	3	8	4	39
**PP2 + I/R**		4				4	8
**Total**	48	32	32	12	32	16	188

The same animals were used for detection of CBF, TTC staining and neurological score. TTC: triphenyl tetrazolium; CBF: cerebral blood flow; TTC: triphenyl tetrazolium chloride; NSS: neurological score; Micro: microcirculation; BMC: brain water content; EM: electron microscope; IHC: immunohistochemistry, WB: western blotting;
